# Internal Consistency, Test–Retest Reliability and Measurement Error of the Self-Report Version of the Social Skills Rating System in a Sample of Australian Adolescents

**DOI:** 10.1371/journal.pone.0073924

**Published:** 2013-09-09

**Authors:** Sharmila Vaz, Richard Parsons, Anne Elizabeth Passmore, Pantelis Andreou, Torbjörn Falkmer

**Affiliations:** 1 School of Occupational Therapy and Social Work, Centre for Research into Disability and Society, Curtin University, Perth, Western Australia, Australia; 2 School of Occupational Therapy and Social Work, Curtin Health Innovation Research Institute, Curtin University, Perth, Western Australia, Australia; 3 School of Occupational Therapy, La Trobe University, Melbourne, Vic. Australia; 4 Rehabilitation Medicine, Department of Medicine and Health Sciences (IMH), Faculty of Health Sciences, Linköping University & Pain and Rehabilitation Centre, UHL, County Council, Linköping, Sweden; 5 Department of Community Health and Epidemiology, Dalhousie University, Halifax, Nova Scotia, Canada; RAND Corporation, United States of America

## Abstract

The social skills rating system (SSRS) is used to assess social skills and competence in children and adolescents. While its characteristics based on United States samples (US) are published, corresponding Australian figures are unavailable. Using a 4-week retest design, we examined the internal consistency, retest reliability and measurement error (ME) of the SSRS secondary student form (SSF) in a sample of Year 7 students (*N* = 187), from five randomly selected public schools in Perth, western Australia. Internal consistency (IC) of the total scale and most subscale scores (except empathy) on the frequency rating scale was adequate to permit independent use. On the importance rating scale, most IC estimates for girls fell below the benchmark. Test–retest estimates of the total scale and subscales were insufficient to permit reliable use. ME of the total scale score (frequency rating) for boys was equivalent to the US estimate, while that for girls was lower than the US error. ME of the total scale score (importance rating) was larger than the error using the frequency rating scale. The study finding supports the idea of using multiple informants (e.g. teacher and parent reports), not just student as recommended in the manual. Future research needs to substantiate the clinical meaningfulness of the MEs calculated in this study by corroborating them against the respective Minimum Clinically Important Difference (MCID).

## Introduction

Social skills include socially acceptable learned behaviours that enable people to interact successfully with others and avoid undesirable responses [1]. These include sharing, initiating relationships, helping, giving compliments, self-control, understanding of others’ feelings, and leadership in group situations [2,3]. The development of social skills is a fundamental task for all [4]. Competence in social skills is a general term of an evaluative nature, used to refer to the quality of an individual’s social skill effectiveness or functionality in a given situation [2]. Social competence in children and adolescents serves as a mechanism for meaningful interactions with others, facilitates the formation of friendships, and the engagement in a range of occupations required by life roles [5]. Positive associations exists between social competence, academic performance, and participation in everyday life activities [6–8]. Unfortunately, not all individuals acquire adequate competence in social skills.

Difficulties in achieving social competence can be due to social skill acquisition or performance deficits [9], and may impede the quality of an individual’s social relationships and adjustment. For example, social competence deficits have been linked to social adjustment problems, such as peer rejection, loneliness, reduced school belongingness, and early withdrawal from school [10,11]. A variety of unfavourable outcomes beyond school, including psychopathology, excessive substance and alcohol use, chaotic lifestyle, limited or absent postsecondary education, and reduced workplace participation have been documented among those with social competence deficits [12–15]. The far-reaching implications of poor social skill development on everyday activity participation underscore the need for practitioners to identify those at risk of disadvantageous outcomes from an early age [3]. Accordingly, reliable measures for assessing social skills and detecting social difficulties in children and adolescents are necessary.

Children’s social behaviour has been found to vary across different settings [16]. Best practice recommends that children’s social skills be assessed in the social environments in which the child functions, with assessment of child, other, and contextual variables as part of the assessment [17]. Routinely, practitioners use observation checklists, interviews, behaviour-rating scales, or socio-metric measures of social status among peers to assess social skills/competence in children and youth [14,18,19]. In order to minimize bias, information is collected across various settings (including home, school, recreational situations) by using a range of informants (including child, parent, teacher, peer, etc.) [14]. Behaviour rating scales have several advantages over other methods of assessment routinely used by health professionals to assess social skills [20]. Behaviour rating scales allow for easy, practical, and time-efficient assessment of a variety of traits and behaviors from multiple sources in multiple settings [19,21–23].

While behaviour rating scales capitalise on the informant’s observations in the child’s natural settings, informant (rater) bias (such as middle-class bias or depression) could confound the findings [24,25]. Empirical investigations support the contention that self-perception and cognitions are the most important predictors of behaviour [26]. An individual occupies a unique position to report on his/her behaviours across different situations, including home, classroom, playground, sports practice [27,28]. Various self-report measures have been successfully used over decades in both research and clinical settings to assess depression [29] and overall functioning [28] in children and youth.

Standardised behaviour rating scales form an important component in the evidence based assessment of social skills [30]. Standardised scales organise information in a systematic and quantifiable manner, and allow for empirical examination of their psychometric properties [31]. The Social Skills Rating System (SSRS) is one such standardised behaviour rating scale that allows for collection of social behaviours under a best-practice model of collecting information via multiple informants in multiple settings. Its multisource approach, intervention linkage, and overall strong evidence for reliability and validity cause it to be recognized as one of the most comprehensive and psychometrically robust of the available norm-referenced behaviour rating scales for use with children and youth both with and without disabilities or chronic illness [20,21,32,33].

Over the past decades, there has been exhaustive research on the teacher and parent versions of the SSRS [11,34–41]. The secondary level student self-report version of the SSRS (SSRS-SSF) has been used to test social competency development programs [42], analyse social support development strategies and assess emotional behaviours and components [43,44]. In Australia, all versions of the SSRS are promoted by the Australian Council of Educational Research (ACER) and have been used by the Australian Institute of Family Studies (AIFS) in the Pathways from Infancy to Adolescence: Australian Temperament Project (ATP) [45]. To date, the psychometric rigor of the SSRS-SSF has not been tested in the Australian setting. Consequently, the present study was undertaken to evaluate the internal consistency, test retest reliability and ME of the SSRS-SSF in an Australian sample. The ME indices presented in this paper will enable clinicians outside the US to precisely determine whether a change in students’ social skills after intervention represents a real behavioural change or not.

## Methodology

### Design and Procedure

A ‘4-week’ test–retest design was used, with time as the only known source of variance [46]. Because of the diversity and number of items in the SSRS-SSF, time required to complete the measurement (25 minutes), and the interval between two administrations (4-weeks), it was assumed that participants would not remember their first responses and that no changes in behaviour would have occurred. A date and time that suited the school was arranged, and the SSRS-SSF was administered by the researcher at each school, using standard protocol [3]. Questionnaires were re-administered by the same researcher, using the same protocol, at the same setting and timing, after a 4-week interval.

### Ethical Clearance

Informed written consent was obtained from school principals, parents and students to participate in this study. In situations where the student declined to participate, even with parental consent, they were not included. Students were made aware that they were not obliged to participate in the study, and were free to withdraw from this study at any time without justification or prejudice.

At all stages, the study conformed to the National Health and Medical Research Council Ethics Guidelines [47]. Full ethics approval was obtained from Curtin University Health Research Ethics Committee (Reference number HR 194/2005).

### Participants

One hundred and eighty seven students agreed to participate in the study, and provided both baseline and 4-week follow- up data. The sample included 102 boys and 85 girls, and the average age of all participants was 12 years and 3 months (*SD* = 3.93 months). These students were selected from five randomly selected public schools from two educational districts of metropolitan Perth, Western Australia. Inclusion was extended to all year 7 students who attended regular classes in these schools.

Sample size adequacy was determined by the guidelines set by Bland and Altman, where the standard error of the within-subject standard deviation (*s*_*w*_), is shown to depend on both number of subjects (*n*), and number of observations per subject (*m*). The 95% confidence interval (CI) for *s*_*w*_ is determined to be *sw* +/ 1.96*s*_*w*_/√(2*n*(*m*-1) [48]. With 2 repetitions (*m* = 2), and requiring that the width of this interval is no more than +/- 0.1s_*w*_ (so that we are confident that we know *s*_*w*_ within 10%), the equation above can be solved for *n*. This minimum sample size is calculated to be *n* = 192. Our sample of 187 students is close to this figure, so that we can be confident that the estimate of *s*_*w*_ that we will obtain will be within 10% of its true (population) value.

### Instrument: The secondary level student self-report version of the SSRS (SSRS-SSF)

The SSRS-SSF assesses 39 social behaviours that parents, teachers or other members of the US community considered important, adaptive and functional to deem students in grades 7-12 socially competent [3]. The listed behaviours are categorised into four social skill domains: assertion; self-control; cooperation; and empathy (referred to as subscales) (Table 1) [3]. The SSRS-SSF assesses student’s perspective of the frequency and importance (social validity) of social behaviour to their relationship with others, using a 3-point scale (Table 2).

**Table 1:** Behaviours measured on each subscale of the SSRS-SSF and example of the rating scale used.

Assertion subscale	Cooperation subscale
**Get attention of opposite gender**	Finish classroom work
**Confident on dates**	Do homework
**Start conversation with opposite gender**	Follow teacher’s directions
**Ask for date**	Ask before using things
**Compliment opposite gender**	Use nice voice
**Make friends**	Use free time
**Start conversation with class members**	Listen to adults
**Active in school activities**	Avoid trouble
**Invite others to join activities**	Ask friends for favours
**Ask adults for help**	
Empathy subscale	**Self-control subscale**
**Understand how friends feel**	Accept punishment from adults
**Listen to friends’ problems**	Avoid trouble
**Say nice things to others**	Do nice things for parents
**Talk over classmates’ problems**	Take criticism from parents
**Smile, wave, or nod**	Control temper
**Ask friends to help with problem**	Ignore classmates’ clowning
**Feel sorry for others**	Ignore classmates’ teasing
**Tell others when they’ve done well**	End fights with parents
**Tell friends I like them**	Compromise with parents or teachers
**Stand up for friends**	Disagree without fighting

**Table 2:** Example of the rating scale used in the SSRS-SSF.

	**How often?**				**How important?**		
Social skill	**Never**	**Sometimes**	**Very Often**		**Not important**	**Important**	**Critical**
I start conversations with classmates	0	1	2		0	1	2

Evidence from past research suggests that the total social skills scale version of the SSRS-SSF (frequency rating) has adequate internal consistency (α = .83) to permit its independent use in samples of multiracial US primary and secondary students with and without disabilities or chronic illnesses [3,49]. Subscale internal consistencies of the SSRS-SSF are insufficient to permit independent use for screening social behavioural difficulties (empathy, α = 0.72-0.73; cooperation, α = 0.66-0.68; self-control, α = 0.68; and assertion, α = 0.67-0.69). The 4-week test retest reliability of each subscale and total social skills scale in past investigations did not meet the benchmarked criteria for reliable use [50] (total social skills scale, *r* = 0.68; empathy, *r* = 0.66; cooperation, *r* = 0.54; assertion, *r* = 0.52; and self-control *r* = 0.52) [3]. ME of the SSRS-SSF total social skills scale score (frequency rating) is reported as +/-6 units at 68, and +/-12 units at 95 percent CIs respectively. The ME of the importance rating scale has not been presented in the manual.

### Data analysis

Data analyses were undertaken using SPSS version 17 and SAS Version 9.2 software packages. Screening of the data, as recommended by Tabachnick and Fidell [51], was undertaken. Only 1.1% of data were missing at scale level. The estimation maximization (EM) algorithm and Little’s chi-square statistic revealed that the data were missing completely at random (MCAR) [51,52]. Standard procedures for missing value replacement and scoring as recommended in the SSRS manual were implemented [3]. Given that the design of this study was to appraise the stability of both the frequency and importance rating scales, subscale and total scores for each rating scale were computed using the rules for the frequency scale. Analyses were performed with gender as a fixed factor, using the same strategy used with the standardisation sample [3]. The following indices were computed:

1 Cronbach’s α: To measure the internal consistency (homogeneity) of the SSRS, based on average inter-item correlations and the number of items.
2 Pearson’s correlation coefficient (*r*): To measure the strength of linear association, or the consistency of position between two sets of data [53].
3 Intraclass correlation coefficient (ICC): A two-way random effects absolute agreement model (ICC_2,1_) was computed [54].
4 The Bland and Altman 95% Limits of Agreement (LOA) and the Coefficient of Repeatability (CR) or the Smallest Real Difference (SRD): The Bland and Altman plot was examined visually to examine heteroscedasticity in the data [55]. The Coefficient of Repeatability (CR) also referred to as the Smallest Real Difference (SRD) was calculated by multiplying the Standard Error of Measurement (SEM) by 2.77 (√ 2 x 1.96) to indicate 95% confidence of a real difference between the true scores (the √ 2 term appears as a result of the difference of the two variances) [55–57]. The SEM is the square-root of the within-subject variance (WSV) [i.e., SEM= √WSV = √ (total variance) (1- ICC)].

## Results

### Internal consistency

An internal consistency analysis was performed calculating Cronbach’s α for each of the four subscales (assertion, cooperation, empathy, and self-control), as well as for the total social skills scale score on the frequency and importance rating scale. Salvia and Ysseldyke’s [58] criteria for ‘acceptable internal consistency for screening purposes’ were used to benchmark estimates as recommended by the SSRS developers [3]. As shown in Tables 3 and 4, the internal consistency of the total social skills scale score (α = 0.87) met the benchmark level. With the exception of the empathy subscale (girls = 0.71, and boys = 0.78), all other subscales had acceptable α-values. On the importance rating form, variability in internal consistency due to gender was noted. The α-value of the total social skills scale score for girls fell below the benchmark (α = 0.78) while that for boys exceeded the benchmark level (α = 0.88). Similarly, lower α-values were identified on the empathy, cooperation, and self-control subscales for girls, all of which were in the moderate category [59]. In the case of boys, the internal consistency estimates (for each subscale and total scale score) met minimal criteria of acceptable value for screening purposes.

**Table 3:** Comparison of measures of reliability for social skills Frequency rating scale.

**Frequency Rating Scale**			**Time 1**		**Time 2**			**Relative and absolute reliability indices**										
	**GENDER**	**N**	**M**	**SD**	**M**	**SD**	**α**	**r_a_**	**ICC_2,1_**	**Mean diff (Bias)**	**SD_diff between subject_**	**t**	**p-value**	**95%LOA (95% CI) LB**	**95%LOA (95% CI) UB**	**Within –subject Variance**	**SEM= √(WSV)**	**CR**
**Assertion**	M	84	13.24	3.11	13.90	3.10	0.89	0.78	0.77	0.66	17.20	2.90	0.005	-3.4 (-4.1 to -2.6)	4.7 (3.9 to 5.5)	2.30	1.52	4.21
	F	74	12.86	3.07	13.27	3.07	0.84	0.72	0.72	0.40	16.22	1.52	0.13	-4.1 (-5.0 to- 3.2)	4.9 (4.0 to 5.8)	2.66	1.63	4.52
**Empathy**	M	98	14.44	2.95	13.95	3.06	0.78	0.62	0.62	-0.49	14.64	-1.86	0.06	-5.6 (-6.5 to -4.7)	4.6 (3.7 to 5.5)	3.49	1.87	5.18
	F	92	16.66	1.93	16.27	2.04	0.71	0.54	0.53	-0.38	6.07	-1.89	0.06	-4.1 (-4.8 to -3.4)	3.4 (2.7 to 4.1)	1.89	1.37	3.81
**Cooperation**	M	96	14.37	2.71	13.92	2.81	0.87	0.78	0.77	-0.45	13.53	-2.39	0.019	-4.1 (-47 to -3.4)	3.2 (2.5 to 3.8)	1.78	1.34	3.70
	F	84	16.35	2.33	16.06	2.65	0.82	0.64	0.63	-0.28	10.17	-1.20	0.23	-4.5 (-5.3 to -3.6)	3.9 (3.1 to 4.7)	2.28	1.51	4.18
**Self-control**	M	92	11.51	2.97	11.84	2.97	0.86	0.67	0.67	0.33	14.75	1.31	0.19	-4.4 (-5.3 to -3.5)	5.1 (4.2 to 5.9)	2.93	1.71	4.75
	F	86	13.65	3.44	13.60	2.97	0.84	0.71	0.70	-0.05	17.60	-0.22	0.82	-4.9 (-5.9 to -4.0)	4.8 (3.9 to 5.7)	3.05	1.75	4.84
**Total Social skills**	M	102	53.53	8.55	53.64	8.72	0.87	0.75	0.76	0.11	130.86	0.18	0.85	-11.8 (-13.8 to -9.7)	12 (9.9 to 14.1)	18.24	4.27	11.84
	F	85	58.33	7.64	58.16	8.08	0.87	0.75	0.75	-0.16	108.30	-0.28	0.78	-11.0 (13.1 to -9.0)	10.7 (8.6 to 12.8)	15.18	3.90	10.80

ICC_2, 1_ Intraclass correlation coefficient: two-way random effect model (absolute agreement definition)

95% LOA LB (95% CI of the LOA) = Bland and Altman 95% Limits of agreement Lower Boundary (95% Confidence intervals of the limits of agreement)

95% LOA UB (95% CI of the LOA) = Bland and Altman 95% Limits of agreement Upper Boundary (95% Confidence intervals of the limits of agreement)

CR = 2.77 × SEM

**Table 4:** Comparison of measures of reliability for social skills Importance rating scale.

**Importance Rating Scale**			**Time 1**		**Time 2**					**Relative and absolute reliability indices**									
	**GENDER**	**N**	**M**	**SD**	**M**	**SD**	**α**	**r_a_**		**ICC_2,1_**	**Mean diff (Bias)**	**SD_diff between subject_**	**t**	**p-value**	**95%LOA (95% CI) LB**	**95%LOA (95% CI) UB**	**Within –subject Variance**	**SEM= √(WSV)**	**CR**
**Assertion**	M		11.46	4.14	11.44	4.18	0.80	0.67		0.67	-0.03	28.96	-0.07	0.95	-6.64 (-7.9 to- 5.3)	6.58 (5.3 to 7.9)	5.60	2.37	6.56
	F	69	11.22	3.41	11.04	3.74	0.81	0.69		0.69	-0.17	16.76	-0.51	0.61	-5.76 (-6.9 to- 4.6)	5.42 (4.2 to 6.6)	3.24	1.80	4.99
**Empathy**	M	97	12.84	3.58	11.60	4.02	0.79	0.66		0.62	-1.23	21.73	-3.83	0.000	-7.40 (-5.8 to- 6.3)	4.94 (3.9 to 6.0)	5.64	2.37	6.58
	F	86	14.45	3.12	13.40	3.87	0.68	0.52		0.48	-1.04	14.29	-2.79	0.006	-7.9 (-9.1 to- 6.6)	5.8 (4.5 to 7.0)	5.94	2.44	6.76
**Cooperation**	M	93	13.65	3.83	12.19	4.27	0.82	0.70		0.65	-1.45	22.43	-4.44	0.000	-7.64 (-8.8 to- 6.5)	4.74 (3.6 to 5.9)	6.13	2.48	6.87
	F	73	15.10	3.23	13.93	3.90	0.67	0.51		0.47	-1.16	16.98	-2.78	0.007	-8.16 (-9.6 to- 6.7)	5.84 (4.4 to 7.3)	6.97	2.64	7.32
**Self-control**	M	83	12.54	3.83	11.94	4.17	0.87	0.77		0.76	-0.67	26.99	-2.25	0.027	-6.04 (-7.1 to- 5.0)	4.70 (3.7 to 5.7)	3.82	1.95	5.42
	F	76	14.25	3.53	13.21	4.15	0.77	0.63		0.59	-1.03	19.22	-2.70	0.009	-7.62 (-8.9 to- 6.3)	5.56 (4.2 to 6.9)	5.57	2.36	6.54
**Total Social skills**	M	101	50.55	13.51	47.91	14.35	0.88	0.79		0.78	-2.64	288.15	-2.94	0.004	-20.38 (-23.4 to -17.3)	15.1 (12.0 to 18.2)	43.51	6.60	18.28
	F	82	53.45	10.85	51.49	13.96	0.78	0.66		0.63	-1.96	212.66	-1.67	0.10	-22.89 (-26.9 to -18.90)	18.97 (14.9 to 23.0)	50.14	7.08	19.63

ICC_2, 1_ Intraclass correlation coefficient: two-way random effect model (absolute agreement definition)

95% LOA LB (95% CI of the LOA) = Bland and Altman 95% Limits of agreement Lower Boundary (95% Confidence intervals of the limits of agreement)

95% LOA UB (95% CI of the LOA) = Bland and Altman 95% Limits of agreement Upper Boundary (95% Confidence intervals of the limits of agreement)

CR = 2.77 × SEM

### Indices of relative reliability

Correlations between the test and retest scores on each subscale and total social skills scale score were estimated using Pearson’s *r* and the ICC _(2, 1)_ statistics. Vincent’s benchmarks were used as the benchmark to interpret Pearson’s *r* and ICC, wherein a value of over 0.90 was considered high, between 0.80 and 0.90 labelled moderate, 0.80 and below insufficient [50]. The 4-week stability correlation for the total social skills scales and subscales (both frequency and importance) did not meet the recommended benchmarks for reliable use.

### ME: Indexed by the Coefficient of Repeatability (CR) or the Smallest Real Difference (SRD)

**Figure 1:**
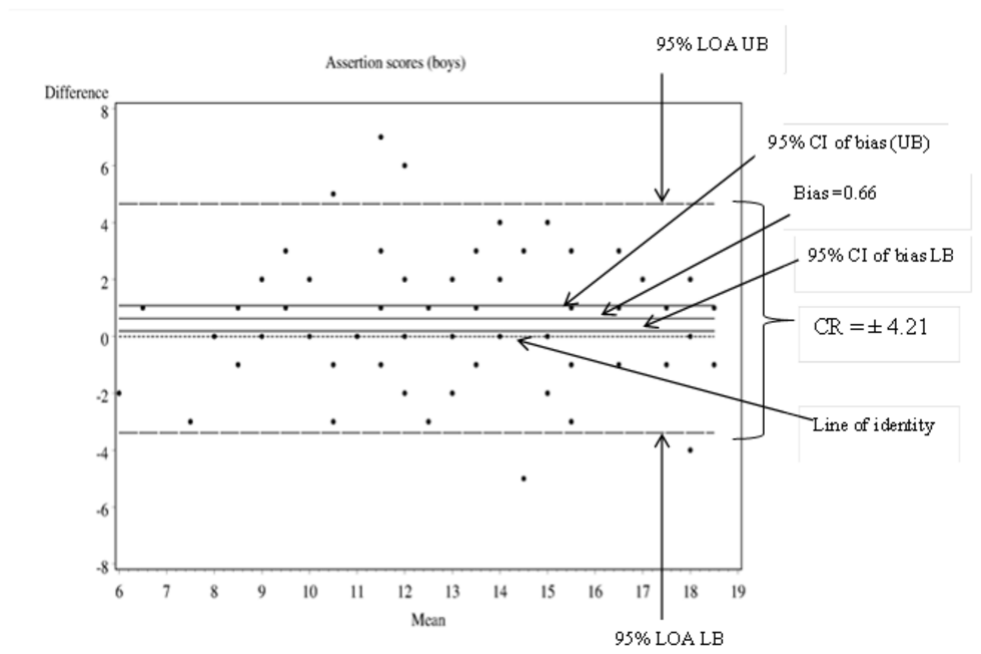
Bland and *Altmandifference* plot using boys' assertion frequency scores as an example.

**Figure 2:**
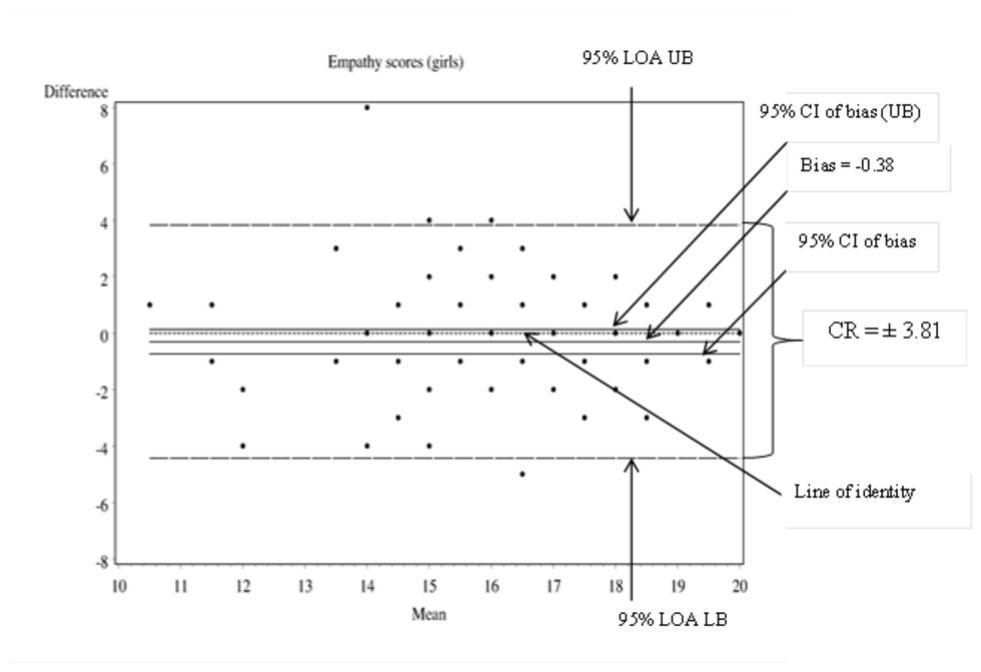
Bland and *Altmandifference* plot using girls’ empathy frequency scores as an example.

The Bland and Altman plot was used to show the 95% upper and lower Limits of Agreement (LOA) which represent the boundaries of ME [55,60]. Following this method, the direction and magnitude of the scatter of difference scores around the zero line were explored by plotting the difference in values against respective mean scores (Figures 1 and 2). The plot of difference against mean scores also allowed investigation of any possible relationship (correlation) between ME and the assumed true value (i.e., the mean value of two methods). To test for heteroscedasticity, the correlation between the differences and the mean of the observations was calculated and tested against the null hypothesis of *r* = 0. Heteroscedasticity was found not to be present on all subscale and total scale scores. In each exploration, the Upper and Lower Limits of Agreement (LOA) bounds and their 95% CIs were spread on either side of zero and met the Bland and Altman criteria to classify the the difference between the two measurements to be due to ME alone [55,61,62]. The repeatability coefficient (CR) also referred to as the Smallest Real Difference (SRD) was computed to assess the ME for each subscale and scale, on the frequency and importance rating systems [56,61,63]. The CR gives the value below which the absolute differences between two repeated social skills scale/subscale scores, in another year 7 Australian student, would lie with 0.95 probability [64].

Figure **1** **.**

**Bland and *Altmandifference* plot using boys’ assertion frequency scores as an example.**

Tables 3 and 4 present the boundaries of true change in social skills on each subscale using frequency and importance ratings. The ME of the total social skills frequency scale for boys (CR = 11.84) was similar to the published figures from the US sample equivalent, while that for girls (CR = 10. 80) was less than the corresponding US estimate of 12 units [3]. Although the ME of the importance rating scale was not presented in the manual, for the current sample of year 7 Australian students, the CR on the importance subscale was wider than that on the frequency subscale.

## Discussion

Standardised tools are increasingly being recognised as an essential component of evidence-based practice. Reliance on these tools places demands on clinicians to understand their properties, strengths and weaknesses, in order to interpret results that influence clinical decisions. This study presents evidence on the internal consistency, test–retest reliability and ME of the secondary level student self-report version of the SSRS (SSRS-SSF), using a sample of grade 7 students from Australia. The self-report version was selected based on the evidence that an adolescent’s perceptions of behaviours is the most reliable marker of psychosocial outcomes [16,27].

The present study found acceptable levels of internal consistency for the total social skills scale score, for both genders (frequency scale). On the importance rating scale, student gender appeared to moderate the internal consistency estimate, with the total scale score for girls falling just short of the benchmarked threshold. Internal consistency estimates of subscales (frequency) suggested better homogeneity in the current sample than that reported in the manual [3]. In the case of the US standardisation sample, none of the subscales (frequency) had homogeneity coefficients above the standard for acceptable use for screening purposes [3,59]. In the case of our Australian sample, all subscales on the frequency form apart from the empathy frequency subscale (across gender) were sufficiently homogenous to permit reliable independent use. On the importance rating scale, however, the empathy, cooperation, and self-control subscales for girls were not found to be homogeneous enough for independent use. Clinically, these findings highlight the need for practitioners in US and Australia to exercise caution while using the less homogenous subscales as independent screeners of the social skills constructs they have been designed to measure.

Pearson’s correlation and the random effects ICC _(2-1)_ were used to assess the 4-week test–retest stability of each subscale and total scale score, on both the frequency and importance rating systems [53]. For the current sample, the Pearson’s *r* and ICC estimates were similar in value, for each subscale and scale score, on both the frequency and importance rating scales. Estimates of all subscale and scale scores (on the frequency and importance rating forms) did not meet the benchmarked criteria for reliable use [50]. The insufficient reliability estimates reported in these studies as well as the SSRS manual suggest that clinicians should avoid using the SSRS-SSF as a sole measure of year 7 students’ social skills.

The CR were computed to assess the ME of the SSRS-SSF subscales and total scales, on the frequency and important rating forms [55,56,63]. The CR includes both systematic and random error in its value and gives the value below which the absolute differences between two repeated social skills scores would lie with 0.95 probability [61,64]. As an example, based on the current study’s findings, clinicians using the SSRS-SSF total social skills scale score (frequency form) with a year 7 Australian youth would need to see a change of at least, ± 11.80 at re-assessment, to be 95% confident that the boy had, in fact, benefited from the intervention. The ME of the total social skills frequency scale was comparable to US norms reported in the manual during scale standardisation [3]. The ME of the total social skills scale score for the current sample (boys = ± 18.28 and girls = ± 19.63) were wider than the equivalent errors on the frequency scale; despite using the same method to compute the scores [3]. Based on the ME indices presented in this study, one could conclude that relative to the frequency rating scale, the importance rating scale of the SSRS-SSF has wider ME.

It is important to recognise that ME estimates of the SSRS-SSF presented in this paper hold limited clinical importance beyond setting the boundaries of the minimal detectable true change [56]. ME does not provide an understanding into whether the change in score is of clinical importance. The latter is determined by the Minimum Clinically Important Difference (MCID) [65], which is decided on clinical grounds (and not based on statistical analysis). The clinical suitability of ME of the SSRS-SSF presented in this study needs to be corroborated against its MCID score to substantiate its clinical relevance. Given past use of the SSRS as a screener of behaviour problems and in treatment effectiveness intervention studies, the research is desirable as clinically meaningful change could be masked if the ME (i.e., the CR in this context) of each subscale and total scale score is wider than the respective MCIDs [57].

The focus of this study was on the reliability of the secondary self-report student version of the SSRS. We recognize that the version of the SSRS used in this study is appropriate for use with children in Grades 7-12. Our explicit focus on Grade 7 children limits the ability to generalize the findings of this study to other grade levels for which this instrument may be used. The overall generalizability of the study’s findings is limited due to the small sample size of the study (*N* = 187) [48]. It is important to note that Pearson’s (r) does not measure agreement, but instead is a measure of how well the data fit a straight line. Despite its limitation, the ICC can be applied to more than two retest administrations. We acknowledge that the Bland and Altman method cannot be cannot be directly applied beyond paired data.

A newer version of the SSRS-SSF called the Social Skills Improvement System-Rating System (SSIS-RS) is in circulation [66]. Preliminary comparability studies of the SSIS-RS against the SSRS in a US sample look promising [67]. Based on the findings of the present study, it is important that researchers assess the ME and MCID of the SSIS-RS in an Australian sample before using it in practice.
